# AMICA1 is a diagnostic and prognostic biomarker and induces immune cells infiltration by activating cGAS-STING signaling in lung adenocarcinoma

**DOI:** 10.1186/s12935-022-02517-x

**Published:** 2022-03-05

**Authors:** Ziyang Feng, Yan Zhang, Min He, Ying Han, Changjing Cai, Shanshan Liu, Ping Liu, Yihong Chen, Hong Shen, Shan Zeng

**Affiliations:** 1 Department of Oncology, Xiangya Hospital, Central South University, Changsha, 410008 Hunan China; 2 Key Laboratory for Molecular Radiation Oncology of Hunan Province, Xiangya Hospital, Central South University, Changsha, 410008 Hunan China; 3 Department of Oncology, Yueyang People’s Hospital, Yueyang Hospital Affiliated to Hunan Normal University, Yueyang, 414000 Hunan China; 4 National Clinical Research Center for Geriatric Disorders, Xiangya Hospital, Central South University, Changsha, 410008 Hunan China

**Keywords:** LUAD, AMICA1, Biomarkers, Bioinformatics, Immune infiltration, cGAS-STING

## Abstract

**Background:**

Adhesion molecule interacting with CXADR antigen 1 (AMICA1), also known as Junction Adhesion Molecule Like (JAML), a recently identified member of the JAMs family, plays a critical role in mediating cancer development and immune cells transmigration. However, AMICA1 has never been reported to be related to the genesis, development and immunotherapy effect of lung adenocarcinoma (LUAD). In this research, we investigated the role of AMICA1 in LUAD through bioinformatic analysis and in vitro experiments.

**Methods:**

Bioinformatic analysis from TCGA and GEO databases were used to investigate the expression level of AMICA1 and the correlation between AMICA1 and clinical parameters in LUAD patients. The LinkedOmics database was analyzed to investigate the co-expression network of AMICA1. TIMER and TISIDB databases were used to analyze the correlation between AMICA1 expression and immune infiltration level. Except for bioinformatic analysis, the AMICA1 mRNA (26 patients) and protein level (6 patients) were also detected by real-time PCR and western blot. The infiltration level of CD8^+^ T cells (15 patients) and PD1^+^ T cells (13 patients) were detected by immunohistochemistry. The diagnostic value of AMICA1 was revealed by receiver operating characteristic (ROC) curves. The Spearman correlation coefficient was used to analyze the correlation between AMICA1 expression and CD8^+^ T cells and PD1^+^ T cells infiltration level.

**Results:**

Bioinformatic data from public database and our data showed that AMICA1 was significantly downregulated in LUAD. Decreased AMICA1 expression in LUAD was associated with higher T stage, M stage and pathological stage. Kaplan–Meier survival analysis indicated that patients with low AMICA1 expression had a worse prognosis. ROC curves showed that AMICA1 had high diagnostic accuracy for LUAD patients. Multivariate Cox analysis further displayed that AMICA1 expression level was an independent prognostic factor for LUAD patients. Moreover, the expression of AMICA1 was significantly different in the immune cells subtype and was obviously linked to immune cells infiltration. In vitro experiments suggested that AMICA1 significantly suppressed the proliferation of LUAD cells and played an important role in activating cGAS-STING signaling.

**Conclusions:**

Our study suggested that AMICA1 might function as a diagnostic and prognostic biomarker and significantly suppressed the proliferation of LUAD cells. Besides, AMICA1 is positively correlated with immune cells infiltration in LUAD, and cGAS-STING signaling might play an important role in the process.

**Supplementary Information:**

The online version contains supplementary material available at 10.1186/s12935-022-02517-x.

## Introduction

Lung cancer is the underlying cause of cancer-linked deaths globally [1]. During the past decades, lung adenocarcinoma (LUAD) has become the dominant subtype of lung cancer [2]. Due to the fact that most LUAD patients are diagnosed at the advanced stage, the 5-year survival rate for lung cancer is still extremely poor in spite of the huge improvements in cancer-related treatment technology [3]. Generally, the 5-year overall survival rate (OS) for LUAD patients is just 15%. Therefore, it is imperative to search for new biomarkers of LUAD. Besides, the great efficacy of immunotherapy has made immune-related biomarkers even more valuable.

The infiltration level of immune cells in tumor microenvironment (TME) plays a cardinal role in tumor initiation, progression, metastasis and treatment resistance [4–6]. Junctional adhesion molecules (JAMs) are members of the immunoglobulin superfamily and they are expressed widely in epithelial cells, endothelial cells, leukocytes, platelets and erythrocytes [7]. The classic members of the JAMs family include JAM-A, -B, and -C, which are widely involved in regulating cell migration and movement [7]. Adhesion molecule interacting with CXADR antigen 1 (AMICA1), also named junctional adhesion molecule-like (JAML), is a recently identified member of the JAMs family, which has been reported to mediate the transmigration of neutrophils and monocytes by interacting with the coxsackie–adenovirus receptor (CAR) [8–10]. Besides, researchers have proved that treatment with anti-AMICA1 antibodies or AMICA1 knockdown can reduce the effectiveness of dendritic cell-based cancer immunotherapy, which means that AMICA1 may function as a potential novel immunotherapy target. However, the correlation between AMICA1 and LUAD genesis, prognosis and immunotherapy effect has never been reported.

Thus, we analyzed LUAD patients’ AMICA1 expression data in TCGA and GEO, and assessed the correlation between AMICA1 expression and LUAD clinical parameters. Besides, we also analyzed the functional networks of AMICA1 and explored its role in tumor immunity. Our findings suggested the possibility of AMICA1 as a new target for diagnosis, prognosis and immunotherapy in LUAD.

## Materials and methods

### Data acquisition and processing

LUAD patients’ gene expression profiles and clinical data were downloaded from TCGA database (https://portal.gdc.cancer.gov/). The samples with a follow-up time less than 30 days were removed. Finally, 437 LUAD samples and 54 non-cancerous adjacent samples were included in this research.

Six LUAD chip datasets GSE116959, GSE32867, GSE43458, GSE32863, GSE75073 and GSE72094 were obtained from GEO database (https://www.ncbi.nlm.nih.gov/geo/). Of the six chip datasets, GSE72094 has detailed clinical prognostic information, so it is used as a validation set to participate in the study, and the other five LUAD datasets were applied to analyze the differential expression of AMICA1. For the probe data, we used the R package hgu133plus2.db to match the probe ID.

Single cell sequence data was obtained from GSE131907 in GEO database.

### LinkedOmics database analysis

The LinkedOmics database (http://www.linkedomics.org/login.php) is a web platform for studying multi-dimensional data sets of 32 cancers in TCGA. Genes co-expression with AMICA1 were presented by volcano figures, heat maps, or scatter plots. Function module can be used to perform GO_BP (Gene Ontology biological process) and KEGG (Kyoto Encyclopedia of Genes and Genomes) pathways analysis.

### TIMER and ESTIMATE database analysis

TIMER (Tumor IMmune Estimation Resource) database (https://cistrome.shinyapps.io/timer/) is a website to analyze immune infiltrates of 32 cancer types. ESTIMATE (Estimation of STromal and Immune cells in MAlignant Tumor tissues using Expression data) database (https://bioinformatics.mdanderson.org/public-software/estimate/) is used to predict the purity of the tumor and the level of stromal/immune cells infiltrating by analyzing tumor tissues gene expression data. ESTIMATE algorithm is based on single sample GSEA and produces three scores: (1) stromal score (that represents the existence of stroma in tumor tissue), (2) immune score (that means the degree of immune cells infiltration in tumor tissue), and (3) estimate score (that infers tumor purity).

### TISIDB database analysis

The TISIDB database (http://cis.hku.hk/TISIDB) integrates 988 reported immune-related anti-tumor genes, high-throughput screening techniques, molecular profling, paracancerous multi-omics data as well as various resources for immunological data obtained from seven public databases. TISIDB enables analyses of associations between AMICA1 and lymphocytes, immunomodulators, and chemokines.

### Patients specimens collection

Primary LUAD tissues and corresponding adjacent non-tumor samples in 26 patients from Xiangya Hospital of Central South University were collected and specimens were immediately stockpiled at − 80 °C. Patients were all diagnosed as LUAD by histopathological examination. Informed consent was obtained from the recruited patients, and the study protocols were approved by the Ethics Committees of the Xiangya Hospitals. All the clinicopathological data were shown in Table 1.

**Table 1:** Clinicopathological parameters of LUAD cohort in Xiangya Hospital

Patient no.	Age (y)	Gender	History of smoking	History of alcohol	Location in lung	Differentiation	T classification	N classification	M classification	TNM stage	Living status and time (days)	AMICA1 relative expression
1	60	Female	No	No	Right upper	Moderately	1a	2	0	IIIA	Living/2024	0.581814025
2	55	Male	No	No	Right upper	Poorly	1	0	0	IA	Living/1759	0.242354344
3	66	Male	Yes	No	Right lower	Poorly	1b	0	0	IA	Living/1457	0.040889893
4	41	Female	No	No	Left lower	Well	2a	0	0	IA	Living/1291	2.790658799
5	67	Male	Yes	No	Right upper	/	2	2	0	IIIA	Dead/1266	2.979596093
6	65	Male	Yes	No	Left upper	Poorly	2a	0	0	IA	Living/1268	2.928418869
7	59	Female	/	/	Left upper	/	2	0	0	IB	Living/1126	4.931463729
8	69	Male	Yes	Yes	Right upper	Poorly	3	0	0	IIB	Living/1285	2.326636974
9	63	Male	Yes	Yes	Right upper	Well–moderately	1c	2	0	IIIA	Living/1399	1.843299506
10	36	Male	No	No	Left upper	/	1c	2	0	IIIA	Living/1338	3.062937628
11	45	Female	/	No	Right lower	/	0	0	1	IV	Living/62	2.133768232
12	61	Male	No	Yes	Right lower	Poorly	1	2	0	IIIA	Dead/1491	1.39632037
13	53	Male	Yes	/	Left lower	Moderately	2	0	0	IB	Living/1674	0.380881822
14	53	Male	Yes	No	Left upper	Moderately–poorly	1b	2	0	IIIA	Living/409	0.169954141
15	66	Male	Yes	Yes	Left upper	/	2	0	0	IB	Dead/1113	3.186495247
16	73	Male	Yes	Yes	Right upper	Poorly	1	0	0	IA	Living/1552	0.627638161
17	60	Male	Yes	No	Left lower	Well-moderately	1	0	0	IA	Living/1491	5.112220858
18	59	Male	/	No	Left upper	Moderately-Poorly	2	0	0	IB	Dead/1583	2.023246747
19	35	Female	No	No	Right lower	Well	4	2	0	IIIB	Living/891	0.359674129
20	74	Male	Yes	Yes	Right lower	Moderately–poorly	4	0	0	IIIA	Living/335	1.82561928
21	58	Female	/	No	Right upper	Moderately	2	0	0	IB	Living/624	0.766687881
22	52	Male	Yes	No	Right lower	Poorly	1	2	0	IIIA	Dead/1552	4.034115729
23	60	Male	No	Yes	Right upper	Moderately	1	0	0	IA	Living/1430	2.322393683
24	32	Female	No	No	Left lower	Moderately	3	3	0	IIIC	Dead/1399	1.324189455
25	42	Male	Yes	Yes	Right upper	Moderately–poorly	1	0	0	IA	Dead/1369	5.418396148
26	60	Female	No	Yes	Right lower	Poorly	2	2	0	IIIA	Living/1004	0.116175814

### Cell lines and cell culture

Human lung cancer cell lines A549, H1437 and H460, human normal pulmonary epithelial cell Beas2B were obtained from the Institutes of Biomedical Sciences (IBS, Shanghai, China) and cultured in RPMI 1640 medium (Gibco, USA) containing 10% fetal bovine serum (FBS) and 1% penicillin/streptomycin (Invitrogen, USA) at 37 °C in a humidified incubator containing 5% CO_2_.

### Quantitative RT-PCR analysis

Total RNA was extracted using Trizol Reagent (Invitrogen, Waltham, MA), and cDNA was synthetized using PrimeScript™ Kit (TaKaRa Bio Inc., Otsu, Japan) following the manufacturer’s instructions. qRT-PCR was performed using a SYBR Green fluorescence-based assay (TaKaRa Bio Inc.) on a ViiA™ 7 RT-PCR system (Applied Biosystems, Carlsbad, CA). The primers for real-time PCR were as follows: AMICA1: forward: GTTTCCCCGCCTGAGCTAAC; reverse: TTCTGGAA GCGCCCAATAGG. GAPDH: forward: TGTGGGCATCAATGGATTT GG; reverse: ACACCATGTATTCCGGGTCAAT. GAPDH was used as reference control. The relative mRNA expression levels were quantified using the 2-ΔΔCt method.

### Western blot

Total protein was extracted with RIPA buffer (P0013B, Beyotime, Shanghai, China) containing protease inhibitor (Thermo Fisher Scientific, Waltham, MA, United States). Protein concentration was determined using the BCA protein assay kit (Beyotime Biotechnology, Shanghai, China). Protein samples were separated by 10% SDS-PAGE and then transferred onto Polyvinylidene fluoride (PVDF) membranes (Millipore, Bedford, MA) under a constant 300 mA. Then, the membranes were incubated with 5% skim milk at room temperature for 1 h and with primary antibodies against AMICA1 (PA5-100845, Thermo Fisher Scientific, Waltham, MA, United States) overnight at 4 °C. After incubating with a Horseradish Peroxidase (HRP)-conjugated secondary antibody for 1 h at 37 °C, the membrane was washed in TBST and prepared for signal detection. The signals were automatically visualized using the ChemiDocXRS + System (Bio-Rad, Hercules, CA) and quantitatively analyzed with Image Lab software (Bio-Rad). GAPDH (abs132004, absin, China) protein expression was used as the internal control.

### Immunohistochemistry

The paraffin‐embedded tissues were subjected to antigen retrieval by microwaving in 0.01 M sodium citrate (pH 6) for 10 min after deparaffinization, hydration and endogenous peroxidase activity elimination, and then incubated overnight at 4 °C with antibody against CD8 (1:100, ab101500, Abcam, Cambridge, UK) or PD1 (1:300, ab137132, Abcam, Cambridge, UK). Subsequently, the slides were incubated with the corresponding secondary antibodies (Zhongshan Goldenbridge Biotechnology, Beijing, China). The number of CD8^+^ and PD1^+^ T cells in five nonoverlapping high-power fields (400×) was counted and the average value was calculated.

### CCK8

Cells were seeded in 96‐well plates at a density of 4000 cells/well in 100 μl of complete medium and grown overnight. After 24, 48, and 72 h certain time point, 10 μl of the CCK‐8 solution (Dojindo, Tokyo, Japan) was added to each well and incubated at 37 °C for 2.5 h. The absorbance per well was measured at 450 nm using a Microplate Reader (BioTek, Winooski, VT). The cell growth curve is generated according to the absorbance of all time points.

### EdU

Cells were seeded in 96‐well plates at a density of 3000 cells/well in 100 μl of complete medium and grown overnight. Then, the cells were maintained with 20 μM EdU labeling medium for 3 h. Subsequently, 4% paraformaldehyde was used to fix the cells, and then the cells were incubated with 100 μl Apollo dye solution (RiboBIO, Guangzhou, China) for 30 min. Then the cells were washed with 0.5% TritonX-100 for 10 min. At last, the cells were stained with DAPI and EdU-positive cells were calculated with ImageJ software.

### Immunofluorescence staining

The cells were washed with ice-cold phosphate-buffered saline (PBS, pH 7.4) and fixed with 4% paraformaldehyde. The cells were then permeabilized with 1% Triton X100, blocked in 2% bovine serum albumin for 30 min prior to incubation with the primary antibodies against TMEM173 (1:100, 66680-1, Proteintech) overnight at 4 °C, and subsequently incubated with Alexa Fluor 488-conjugated secondary antibodies for 1 h at room temperature (1:1000, ab150113, Abcam, Cambridge, UK). Then the cells were incubated with DAPI for 10 min. Images were captured using a N2-DMi8 microscope (Leica, Wetzlar, Germany).

### Statistical analysis

Differential expression of AMICA1 on LUAD tissues were determined using Wilcoxon single rank test. A received operating characteristic (ROC) curve was applied to assess the diagnostic value of AMICA1, with the area under the ROC curve used as the diagnostic value. The Wilcoxon test and Kruskal test were applied to analyze the correlation between AMICA1 expression level and different clinicopathological features in LUAD patients. Univariate Cox analysis was used to screen potential prognostic factors, and multivariate Cox analysis was used to verify the effect of AMICA1 expression on survival along with other clinical variables. Pearson’s correlation coefficient was used to analysis the co-expression gene with AMICA1. Spearman correlation coefficient was used to analysis the correlation between AMICA1 and immune cells and molecules. T test was used to compare differences among groups. SPSS 17.0 was used for data analyses. Statistically significant was set on P-value < 0.05.

## Results

### AMICA1 is down-regulated in LUAD and may function as a diagnostic biomarker

**Fig. 1:**
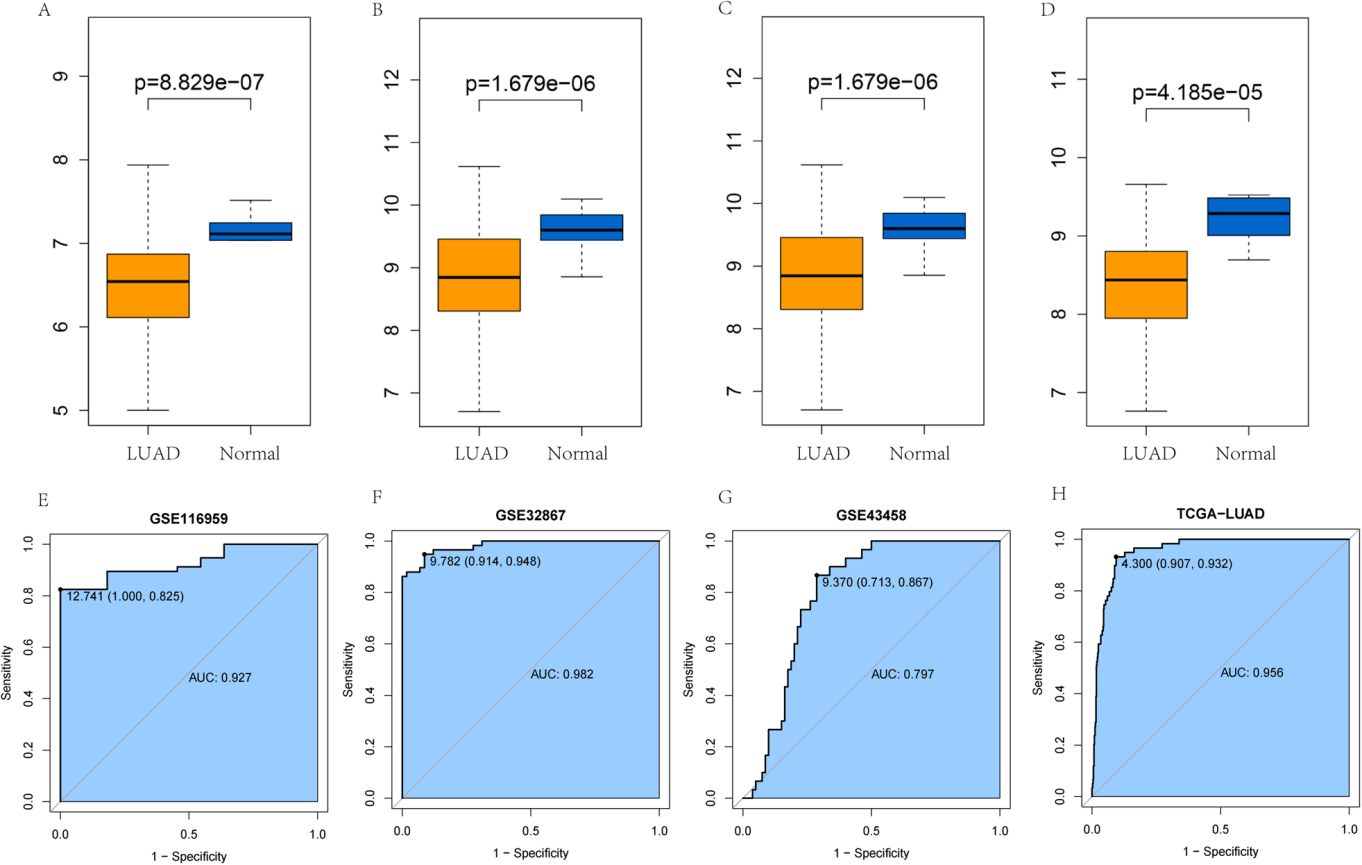
The expression level and diagnostic value of AMICA1 in LUAD. **A**–**D** The expression of AMICA1 in LUAD, including GSE116959 (T = 57, N = 11, P < 0.001), GSE32867 (T = 145, N = 144, P < 0.001), GSE43458 (T = 80, N = 30, P < 0.001) and TCGA database (T = 437, N = 54, P < 0.001). **E**–**H** ROC curve of GSE116959 (AUC = 0.927, Sensitivity = 0.825, Specificity = 1), GSE32867 (AUC = 0.982, Sensitivity = 0.948, Specificity = 0.914), GSE43458 (AUC = 0.797, Sensitivity = 0.867, Specificity = 0.713) and TCGA database (AUC = 0.956, Sensitivity = 0.932, Specificity = 0.907)

We first assessed AMICA1 mRNA expression level in GSE116959 (T = 57, N = 11, P < 0.001, Fig. 1A), GSE32867 (T = 145, N = 144, P < 0.001, Fig. 1B), GSE43458 (T = 80, N = 30, P < 0.001, Fig. 1C) and TCGA database (T = 437, N = 54, P < 0.001, Fig. 1D), and the results obtained showed that, compared with normal tissues, LUAD patients’ AMICA1 expression was obviously lower. We further investigated the AMICA1 expression level in LUAD tissues and matched paracancerous non-cancerous tissues in GSE32863 (T = 60, N = 60, P < 0.001, Additional file 1: Fig. S1A), GSE75037 (T = 83, N = 83, P < 0.001, Additional file 1: Fig. S1B) and TCGA (T = 49, N = 49, P < 0.001, Additional file 1: Fig. S1C) database through Wilcoxon single rank test, and discovered that the AMICA1 expression was also significantly decreased in LUAD tissues. These findings implied that AMICA1 may play an inhibitory role in the LUAD development. Then, we further investigated the value of AMICA1 in LUAD diagnosis. The ROC curve of GSE116959 (Fig. 1E), GSE32867 (Fig. 1F), GSE43458 (Fig. 1G) and TCGA database (Fig. 1H) showed that AMICA1 can function as a potential diagnostic marker for LUAD, the AUC values were 0.927, 0.982, 0.797 and 0.956, respectively. And the sensitivity was 0.825, 0.948, 0.867, 0.932 and the specificity was 1.000, 0.914, 0.713 and 0.907, respectively. As for the paired LUAD and paracancerous non-cancerous tissues, that is GSE32863 (Additional file 1: Fig. S1D), GSE75037 (Additional file 1: Fig. S1E) and TCGA database (Additional file 1: Fig. S1F), we also calculated the AUC values, they were 0.982, 0.991 and 0.952, respectively. The sensitivity was 0.948, 0.952, 0.932 and the specificity was 0.914, 0.964 and 0.881, respectively. All of these results showed that AMICA1 was down-regulated in LUAD tissue and may function as a biomarker for LUAD diagnosis.

### Correlations between AMICA1 expression and clinicopathological parameters in LUAD patients

**Fig. 2:**
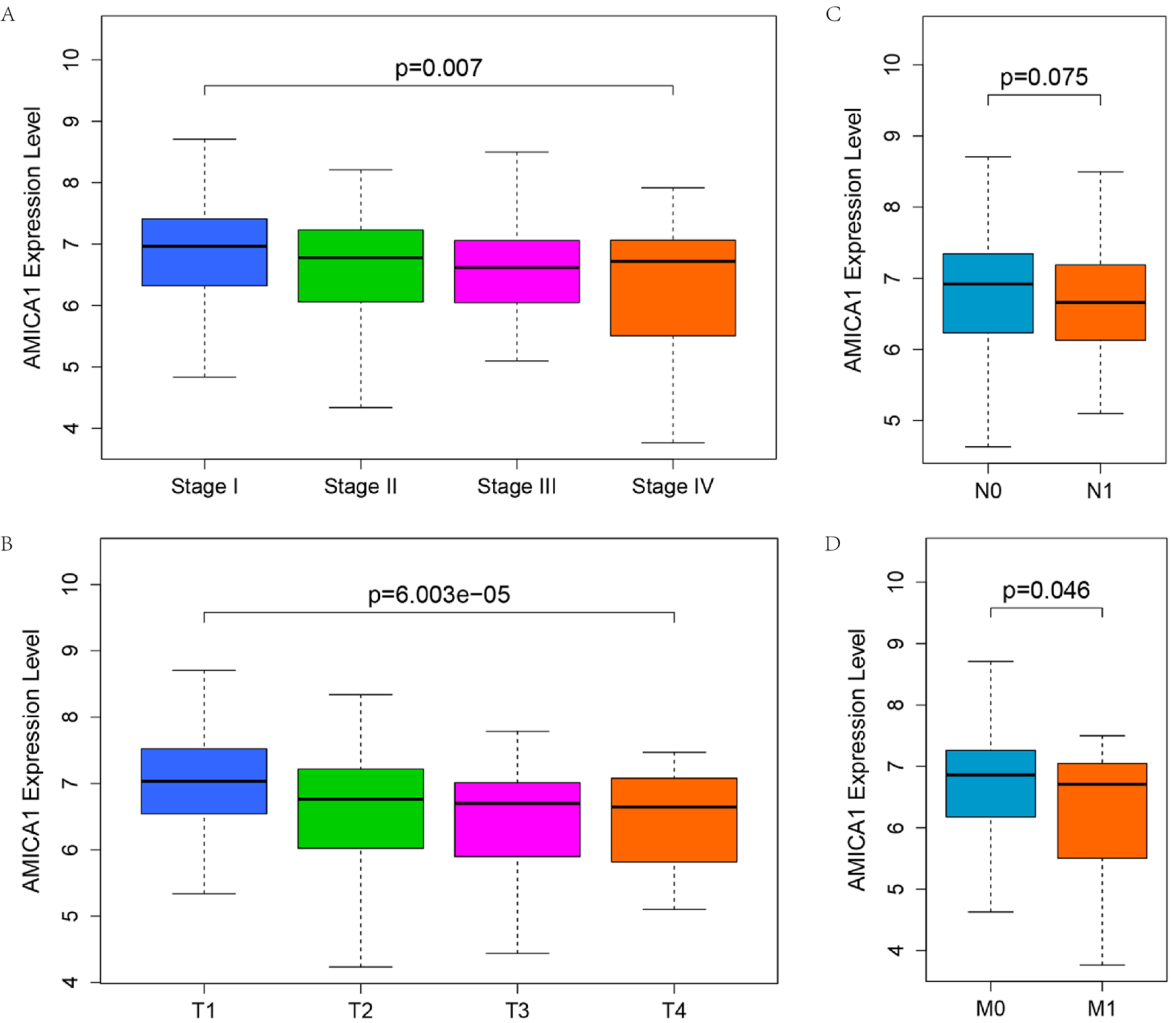
Correlation of AMICA1 expression level with TNM stage. **A** TNM stage (P = 0.007); **B** T stage (P < 0.001); **C** N stage (P > 0.05); **D** M stage (P = 0.046)

Since AMICA1’s function in LUAD is still unclear, it is necessary to further explore the connections between the expression level of AMICA1 and the clinical parameters in LUAD patients. Thus, the Wilcoxon test and Kruskal test were applied to analyze the correlation between AMICA1 expression level and different clinicopathological features in LUAD patients. TNM stage is the most widely used method for tumor stage. T, N, M represents the status of primary tumor, lymph-node metastasis and distant metastasis, respectively. The detailed TNM stage can be seen in the eighth edition IASLC lung cancer stage project [11–13]. TCGA data showed that AMICA1 expression was linked to T stage (P < 0.001, Fig. 2B), M stage (P = 0.046, Fig. 2D) and TNM stage (P = 0.007, Fig. 2A) of LUAD. These results revealed that AMICA1 was significantly decreased in advanced LUAD patients.

Furthermore, we used Cox regression to analyze the prognostic role of AMICA1 in LUAD. The univariate analysis showed that low AMICA1 expression was associated with worse overall survival (OS) (P = 0.006, Additional file 2: Fig. S2A). Besides, as expected, the clinical parameters, such as advanced T stage (P < 0.001), N stage (P < 0.001), M stage (P = 0.019) and TNM stage (P < 0.001), were all related to worse OS (Additional file 2: Fig. S2A). To further verify AMICA1’s prognostic value in LUAD, multivariate analysis was performed. The result obtained revealed that only AMICA1 expression (P = 0.019) and TNM stage (P = 0.006) were independently related to OS (Additional file 2: Fig. S2B), which means that the role of AMICA1 in evaluating patients' clinical prognosis is superior to T stage, N stage and M stage.

### AMICA1 is related to immune infiltration level and LUAD prognosis

**Fig. 3:**
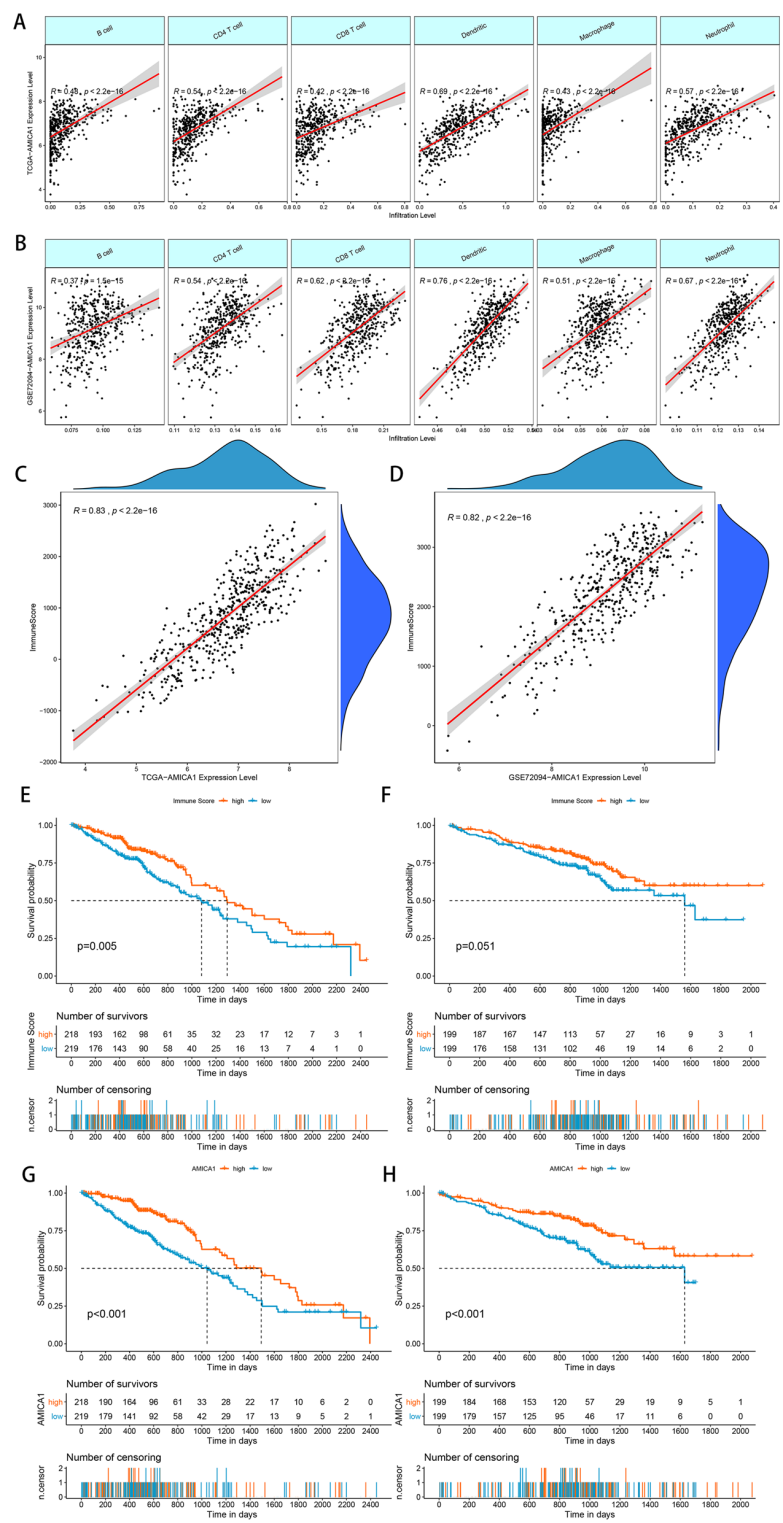
Associations of AMICA1 expression with immune infiltration level in LUAD. **A**, **B** AMICA1 expression levels in the TCGA-LUAD and GSE72094 dataset had a significant positive association with the infiltration level of B cells, CD4^+^ T cells, CD8^+^ T cells, dendritic cells, macrophages and neutrophil. **C**, **D** The expression levels of AMICA1 had a significant positive relation with the immune score of LUAD samples based on the ESTIMATE algorithm in the TCGA-LUAD and GSE72094 datasets. **E**, **F** Kaplan–Meier survival curves analysis showed that patients with high immune scores had higher overall survival time in the TCGA-LUAD and GSE72094 dataset. **G**, **H** Kaplan–Meier survival curves analysis showed that patients with high AMICA1 expression level had higher overall survival time in the TCGA-LUAD and GSE72094 dataset

Next, we explored whether the expression level of AMICA1 was linked to various immune cell infiltration in LUAD from TIMER database. Pearson correlation analysis displayed a significantly positive connection between AMICA1 expression and B cell (R = 0.48, P < 0.001), CD4^+^ T cells (R = 0.54, P < 0.001), CD8^+^ T cells (R = 0.42, P < 0.001), dendritic cells (R = 0.69, P < 0.001), macrophages (R = 0.43, P < 0.001) and neutrophil (R = 0.57, P < 0.001) (Fig. 3A). The positive correlations between AMICA1 expression and these immune cells in the TCGA-LUAD dataset were also well confirmed in GSE72094 dataset (Fig. 3B).

We then used the ESTIMATH algorithm to analyze whether AMICA1 expression was associated with the total level of immune cells infiltration in LUAD. The results obtained showed a positive connection between the expression level of AMICA1 and immune score in both TCGA (P < 0.001, Fig. 3C) and GEO LUAD datasets (P < 0.001, Fig. 3D). Moreover, LUAD patients with high immune scores have a better OS (Fig. 3E and F), which was consistent with the prognostic results of AMICA1 (Fig. 3G and H).

To further broaden the cognition of the correlation between AMICA1 and immune infiltration, we analyzed the connections between AMICA1 and tumor-infiltrating lymphocytes (TILs), immunomodulators, chemokines and related receptors, and respectively listed the top six related cells and molecules. Spearman associations test between AMICA1 expression and various immune signatures were obtained from the TISIDB database. Additional file 3: Fig. S3A (left) shows the correlations between AMICA1 expression and 28 TILs abundance in 30 kinds of cancers. The results obtained showed that AMICA1 was related to many TILs in LUAD, the top six TILs (Additional file 3: Fig. S3A right) were respectively macrophage (r = 0.754, P < 0.001), CD8^+^ effector memory T cells (TEM_CD8) (r = 0.744, P < 0.001), myeloid-derived suppressor cells (MDSCs) (r = 0.735, P < 0.001), mast cells (r = 0.734, P < 0.001), follicular helper T cells (Tfhs) (r = 0.726, P < 0.001) and immature B cells (Imm_B cells) (r = 0.691, P < 0.001). Additional file 3: Fig. S3B–F shows the correlations between AMICA1 and 24 immunoinhibitors, 45 immunostimulators, 21 MHC molecules, 41 chemokines and 18 related receptors, respectively.

Finally, we analyzed the expression of AMICA1 in infiltrating immune cells subtype of LUAD tissues with single-cell sequence data from GSE131907 (neutrophils were not recovered in the experimental process because of technical reasons). All of the patients were treatment-naïve and were divided into early- and advanced- stages. The early-stage was defined as the patients without lymph nodes and distant metastasis. We found out that AMICA1 was mainly expressed in myeloid cells in early- and advanced- stage LUAD tissues (Early-stage LUAD: Additional file 4: Fig. S4A, B, Advanced-stage LUAD: Additional file 4: Fig. S4E, F). Then, we further analyzed the expression of AMICA1 in different myeloid cells subtypes, including monocytes, macrophages (mo-Mac, Alveolar Mac and Pleural Mac) and dendritic cells (CD1c^+^ DCs, CD207^+^ CD1a^+^ LCs, CD163^+^ CD14^+^ DCs, Activated DCs, CD141^+^ DCs and pDCs). Interestingly, the expression of AMICA1 is significantly decreased in mo-Mac compared to other myeloid cell subtypes, including monocytes, alveolar Mac and aleural Mac and is significantly increased in CD1^+^ DCs compared to other DCs subtypes (Early-stage LUAD: Additional file 4: Fig. S4C, D, Advanced-stage LUAD: Additional file 4: Fig. S4G, H). Different from alveolar Mac and aleural Mac, Mo-Mac are monocyte-derived macrophages and could create an immunosuppressive microenvironment [11], which means that decreased AMICA1 expression might play an important role in the formation of mo-Mac and immunosuppression.

### AMICA1 co-expression networks in LUAD

**Fig. 4:**
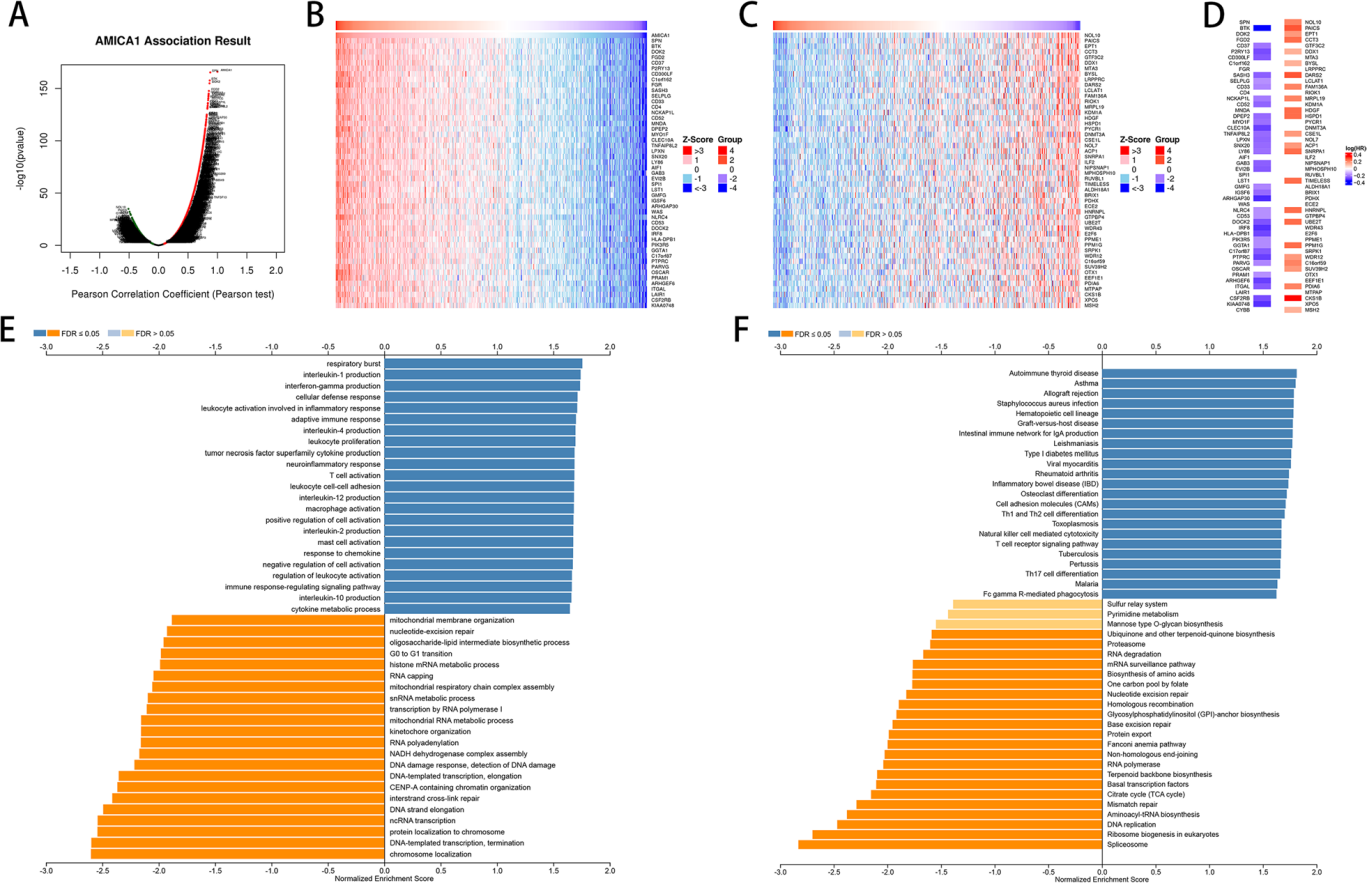
AMICA1 co-expression genes in LUAD. **A** The whole AMICA1 highly associated genes identified by Pearson test in LUAD cohort. **B**, **C** Heat maps showing top 50 genes positively and negatively related to AMICA1 in LUAD. Red shows positively connected genes and blue demonstrates negatively correlated genes. **D** Survival map of the top 50 genes positively and negatively associated with AMICA1 in LUAD. **E**, **F** Significantly enriched GO annotations and KEGG pathways of AMICA1 in LUAD cohort

To further explore AMICA1’s biological significance in LUAD, AMICA1 co-expression network was investigated through the function module of LinkedOmics. Volcano plots in Fig. 4A show that 3207 genes (dark red dots) were positively related to AMICA1, and 1292 genes (dark green dots) were negatively related. Figures 4B and C show respectively the top 50 significant genes that are positively and negatively related to AMICA1. Notably, in the top 50 significantly positive genes, there were 36 genes with a low hazard ratio (HR) (P < 0.05), which means that they may function as the low-risk genes like AMICA1. In contrast, there were 24/50 genes with high a HR (P < 0.05) in the top 50 negatively significant genes (Fig. 4D).

Besides, GO-BP term annotation by GSEA demonstrated that AMICA1 co-expressed genes join mainly in respiratory burst, interleukin-1 production, interferon-gamma production, cellular defense response, leukocyte activation involved in inflammatory response, adaptive immune response, interleukin-4 production, leukocyte proliferation, tumor necrosis factor superfamily cytokine production and neuroinflammatory response, etc. (Fig. 4E). And KEGG pathway analysis illustrated enrichment in autoimmune thyroid disease, asthma, allograft rejection, staphylococcus aureus infection, hematopoietic cell lineage, graft-versus-host disease, intestinal immune network for IgA production, leishmaniasis, type I diabetes mellitus and viral myocarditis, etc. (Fig. 4F).

These results show a wide influence of AMICA1 on the immune response and prognosis in LUAD patients.

### Validation of AMICA1 expression and the correlation with immune infiltration

**Fig. 5:**
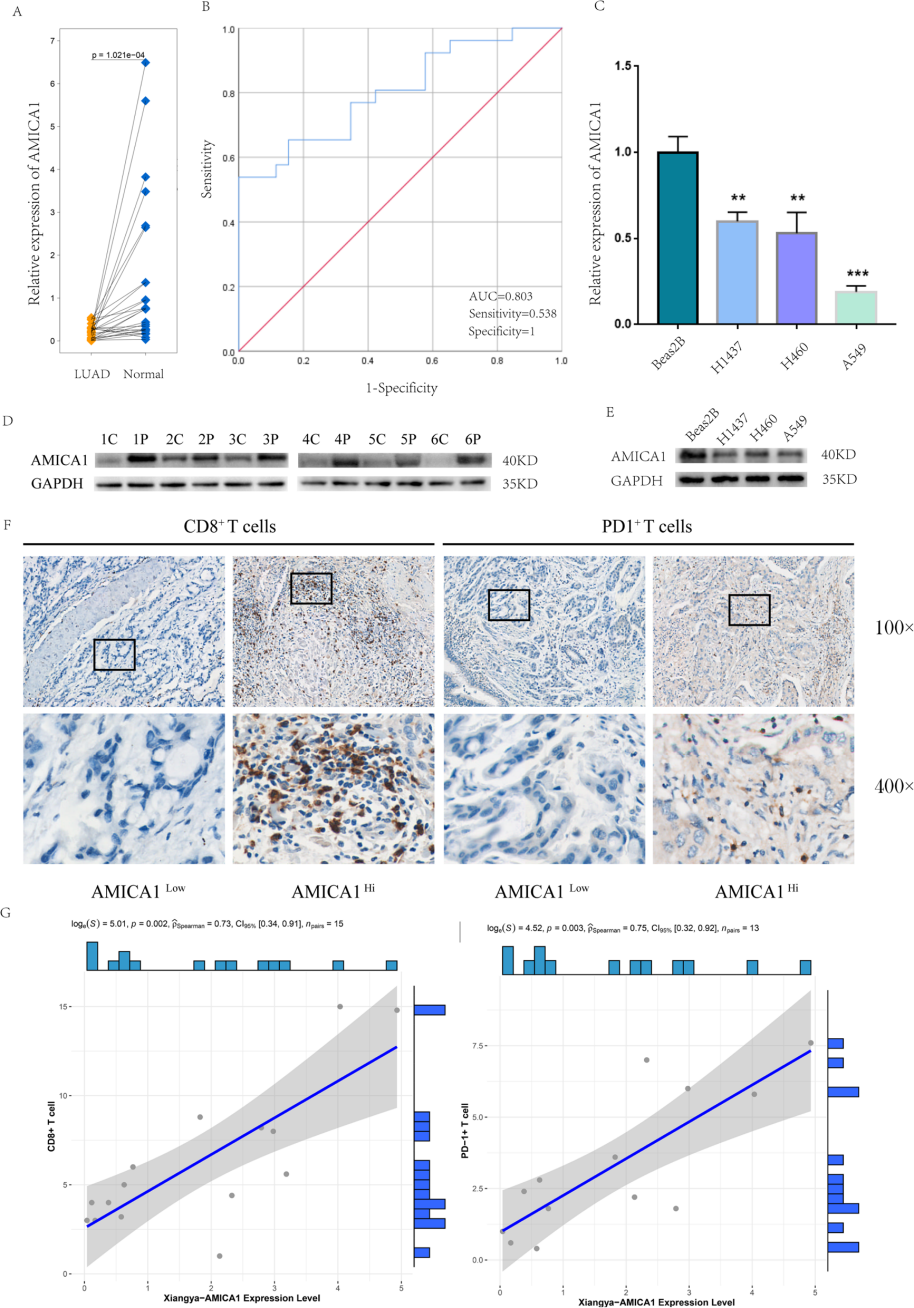
Validation of AMICA1 expression and the correlation with immune infiltration. **A**, **B** AMICA1 mRNA expression level and ROC curve in 26 pairs of LUAD tumor tissues and paracancerous non-tumor tissues. **C** AMICA1 mRNA expression level in lung cancer cell lines (A549, H1437, H460) and human normal pulmonary epithelial cell line (Beas2B). **D** AMICA1 protein expression level in six pairs of LUAD cancer tissues and paracancerous non-tumor tissues (C: cancer tissues P: paracancerous non-tumor tissues). **E** AMICA1 protein expression level in lung cancer cell lines (A549, H1437, H460) and human normal pulmonary epithelial cell line (Beas2B). **F**, **G** AMICA1 expression was positively correlated with the infiltration of PD1^+^ T cells and CD8^+^ T cells

To further confirm the above results, we analyzed AMICA1 mRNA and protein expression levels in LUAD tissues and adjacent non-tumor tissues and several lung cancer cell lines (A549, H1437 and H460) by qRT-PCR and western blotting. Besides, the CD8^+^ T cells and PD1^+^ T cells infiltration levels were detected via immunohistochemistry (IHC). The results obtained show that the mRNA and protein level of AMICA1 were both significantly decreased in LUAD tissues when compared with adjacent non-tumor tissues (Fig. 5A and D). And the ROC curves revealed that AMICA1 can function as a diagnostic marker with AUC = 0.803, Sensitivity = 0.538 and Specificity = 1 (Fig. 5B). Besides, the mRNA and protein level of AMICA1 were both significantly decreased in A549, H1437 and H460 lung cancer cell lines when compared with human normal pulmonary epithelial cell Beas2B (Fig. 5C and E). The IHC results and spearman correlation test confirmed that the expression level of AMICA1 was positively correlated with the infiltration levels of CD8^+^ T cells (P = 0.002) and PD1^+^ T cells (P = 0.003, Fig. 5F, G).

### Overexpression of AMICA1 suppressed the proliferation of LUAD and activated cGAS-STING signaling

**Fig. 6:**
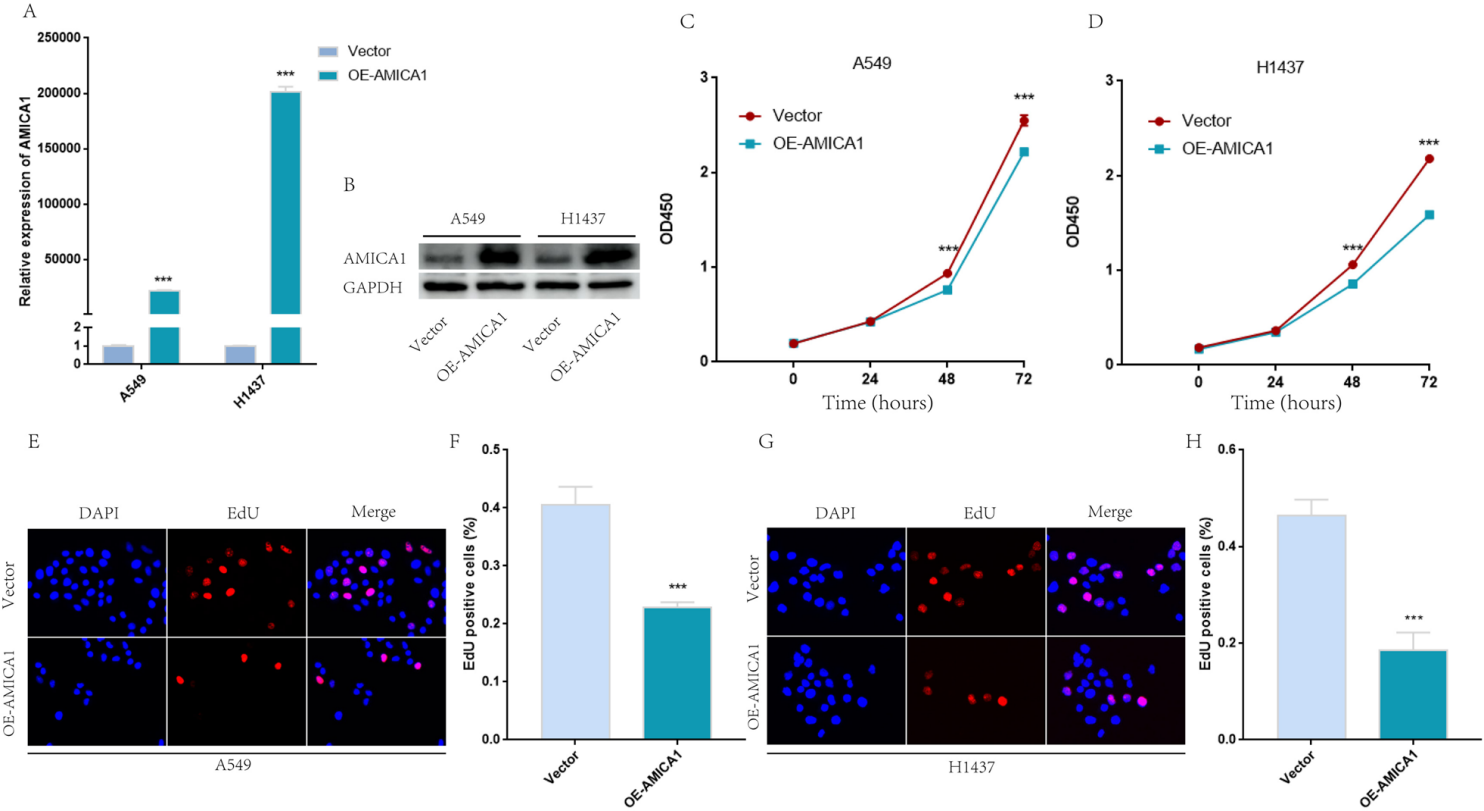
Overexpression of AMICA1 suppressed the proliferation of LUAD cell. **A**, **B** The overexpression efficiency of AMICA1 in A549 and H1437 cells. **C**, **D** CCK8 suggested that AMICA1 suppressed the proliferation of A549 and H1437 cell. **E**–**H** EdU suggested that AMICA1 suppressed the proliferation of A549 and H1437 cell

**Fig. 7:**
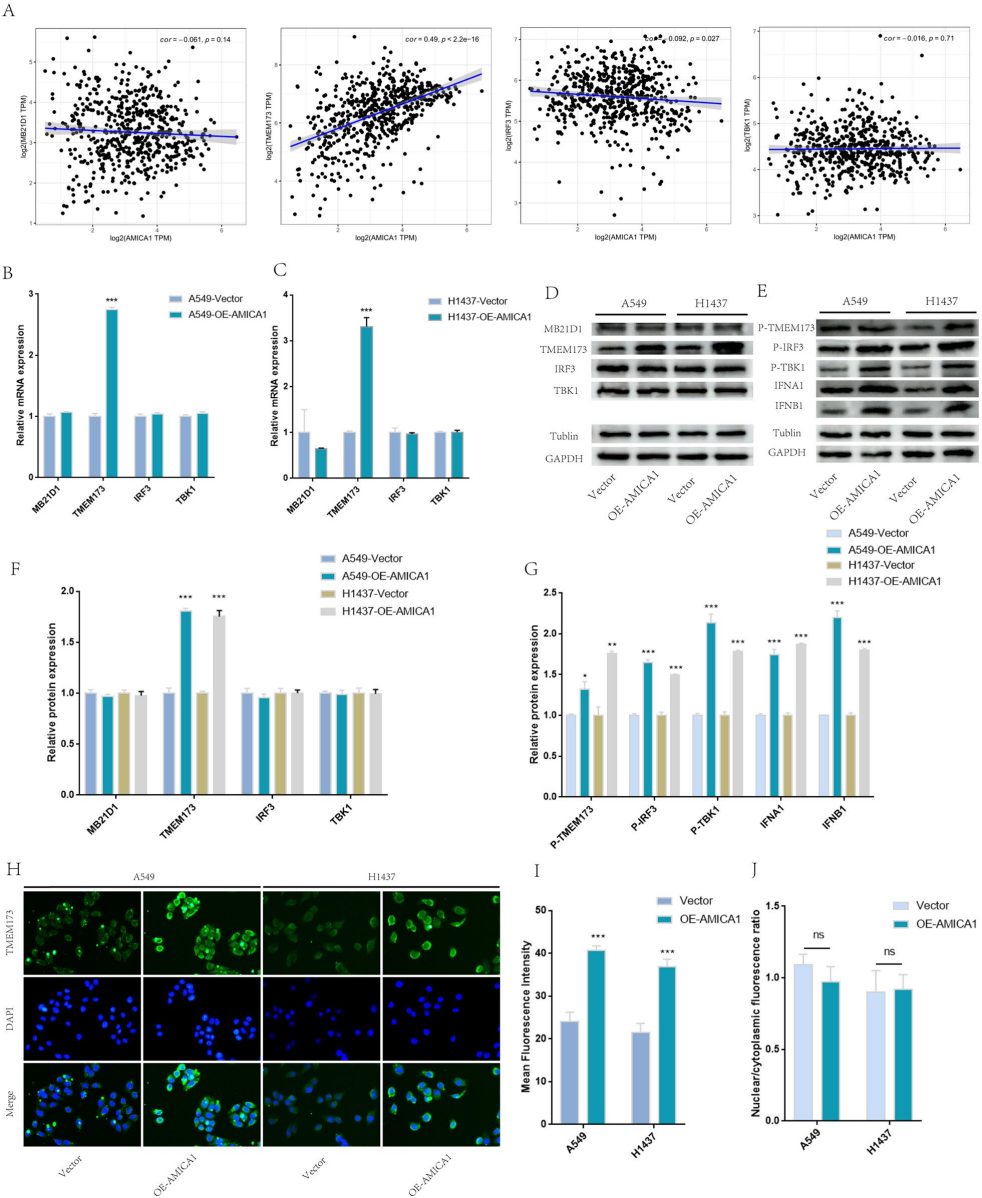
Overexpression of AMICA1 activated cGAS-STING signaling and promoted expression of type I interferon. **A** The correlation between AMICA1 and cGAS-STING signaling mRNA in LUAD tissues. **B**, **C** Over expression of AMICA1 promoted the expression of TMEM173 mRNA. **D** and **F** Over expression of AMICA1 promoted the expression of TMEM173 protein. **E** and **G** Over expression of AMICA1 activated cGAS-STING signaling and promoted expression of type I interferon. **H**, **I** Immunofluorescence staining of TMEM173 in A549 and H1437 cells. **J** AMICA1 had no influence in the location of TMEM173

To determine the biological role of AMICA1 in LUAD proliferation, we overexpressed AMICA1 in A549 and H1437 cell line, the overexpression efficiency was confirmed by qRT-PCR and western blot (Fig. 6A, B). The results of CCK8 (Fig. 6C, D) and EDU (Fig. 6E, H) suggested that overexpression of AMICA1 significantly suppressed the proliferation ability of A549 and H1437 cells. Besides, given that cyclic GMP-AMP synthase and stimulator of interferon genes (cGAS-STING) signaling is important to immune infiltration of LUAD, we investigated the correlation between AMICA1 and cGAS-STING signaling from TCGA database, including MB21D1, TMEM173, IRF3 and TBK1. The results obtained show that the mRNA expression level of AMICA1 in LUAD tissues was positively correlated with TMEM173 (P < 0.001, cor = 0.49, Fig. 7A) and IRF3 (P = 0.027, cor = − 0.092, Fig. 7A). Our results showed that overexpression of AMICA1 could increase the expression of TMEM173 mRNA level (Fig. 7B, C) and protein level (Fig. 7D and F), but had no influence on the expression of MB21D1, IRF3 and TBK1. The result of immunofluorescence also showed that TMEM173 was significantly up-regulated after the overexpression of AMICA1 (Fig. 7H, I). Besides, we further analyzed the nuclear/cytoplasmic fluorescence ratio and found that AMICA1 had no influence in the location of TMEM173 (Fig. 7J). Given that the phosphorylation of TMEM173 is necessary for the activation of cGAS-STING signaling, we then detected the phosphorylation level of cGAS-STING signaling. The results obtained show that the phosphorylation levels of TMEM173, IRF3 and TBK1 were up-regulated in A549 and H1437 cell lines (Fig. 7E and G). Besides, the expression of type I interferon was up-regulated as well (Fig. 7E and G).

## Discussion

Nowadays, the early detection of lung cancer mainly includes imaging examination and serum markers, such as CA125, CEA, NSE, SCC and CYFRA21-1. In our study, we found out that the mRNA and the protein level of AMICA1 were both significantly decreased in LUAD tissues when compared with adjacent non-tumor tissues. And the TCGA, GEO database and our cohort showed that AMICA1 can function as a diagnostic biomarker. Besides, AMICA1 is correlated with the TNM stage and the worse prognosis of LUAD. And our in-vitro experiments also suggested that AMICA1 is significantly down-regulated in A549, H460 and H1437 cells when compared with normal pulmonary epithelial cell Beas2B, and the overexpression of AMICA1 significantly suppressed the proliferation of A549 and H1437 cells. All of these results suggest that AMICA1 may function as a tumor suppressor gene and play an important role in the diagnosis and prognosis of LUAD.

Tumor cells do not grow in isolation but exist in the complex TME. Generally, TME is subdivided into extracellular matrix (ECM), stromal cells and immune cells [14]. The infiltration level of immune cells is closely related to the effectiveness of immunotherapy. According to the immune infiltration level, tumors are classified as “hot” (highly infiltrated, immunoscore I4) and “cold” (non- infiltrated, immunoscore I0) tumors [15]. Numerous studies have focused on the transformation of “cold” tumors and “hot” tumors, because the latter are always more sensitive to immunotherapy. In our study, we found out that AMICA1 is positively related to immune score and the infiltration of several immune cells according to TIMER database, including B cells, CD4^+^ T cells, CD8^+^ T cells, dendritic cells, macrophages and neutrophils. Except for immune cells, we found out that AMICA1 is also positively correlated with the expression of immune modulators, chemokines and related receptors. What’s more, the GSEA and KEGG analysis of AMICA1 co-expression gene showed that AMICA1 may join in several immune-related processes, including interleukin-1 production, interferon-gamma production, leukocyte activation involved in inflammatory response, adaptive immune response, interleukin-4 production and tumor necrosis factor superfamily cytokine production. And through in-vitro experiments, we further verified that AMICA1 is indeed related to the infiltration of CD8^+^ T cells and PD1^+^ T cells. CD8^+^ T cells, as one of the most important effector cells, play important roles in clearing intracellular pathogens and tumor [15]. And anti-PD1 or PD-L1 has become the most classical treatment of immunotherapy. Clinically, by blocking this interaction with anti-PD-1 and anti-PD-L1 antibodies, we can not only promote the proliferation of T cells but also restore their cytotoxic responses against tumor cells [16, 17]. In terms of the mechanism, we found out that overexpression of AMICA1 activated cGAS-STING signaling and promoted the expression of type I interferon. The cGAS-STING pathway could induce the expression of type I interferon to activate antitumor immunity in DC cells and tumor cells [18, 19]. Type I IFN plays a crucial role in promoting the migration and activation of immune cells, including DCs, T cells, and NK cells [20]. Thus, our studies suggest that AMICA1 is positively correlated with the immune infiltration of LUAD, and cGAS-STING signaling might play an important role in the process.

Besides, through single-cell sequence data analysis, we further found out that the expression of AMICA1 is significantly decreased in mo-Mac when compared with monocytes, alveolar Mac and aleural Mac. Different from alveolar Mac and aleural Mac, Mo-Mac is monocyte-derived macrophages and could create an immunosuppressive microenvironment [11], which means that decreased AMICA1 expression might play an important role in the formation of mo-Mac and immunosuppression.

On the other hand, the expression and function of AMICA1 in gastric cancer is contradictory to that in LUAD [21]. In 2021, Yuying Fang et al. found out that AMICA1 expression was higher in gastric cancer tissues than para-carcinoma tissues. High expression of AMICA1 in gastric cancer tissues was related to worse differentiation, local lymph node metastasis, deeper infiltration, and worse stage. And the in vitro experiments also showed that the silence of AMICA1 inhibited GC cell proliferation and migration, and AMICA1 overexpression had an opposite function. In mechanism, they found out that the silence of AMICA1 can significantly inhibit p38 phosphorylation. However, our results show that AMICA1 was down-regulated in LUAD and the high expression of AMICA1 is correlated to a better prognosis. Besides, Christel Moog-Lutz et al. found out that AMICA1 mRNA was up-regulated in all-trans retinoic acid-treated acute promyelocytic leukemia cells, and the mRNA is also up-regulated in myeloid leukemia cells during induced differentiation through granulocytic and monocytic pathways [17], but the mechanism is still unclear. All of these studies provide new insights into the function of AMICA1 in a malignant tumor, which means that the mechanism needs to be further studied.

There are still some limitations to our study. Firstly, in our in vitro experiments, we just detected the expression of AMICA1 in LUAD and adjacent non-tumor tissues rather than in serum samples. To a certain extent, it will limit the clinical application. Secondly, the mechanisms of the correlation between AMICA1 and LUAD prognosis and immune infiltration were still unclear and require further research. Thirdly, the sample size was relatively small, and expanding the sample size may make the results more accurate. We will try to solve these problems in our follow-up study.

## Conclusion

Our study suggests that AMICA1 might function as a diagnostic and prognostic biomarker and it significantly suppressed the proliferation of LUAD cells. Besides, AMICA1 is positively correlated with immune cells infiltration in LUAD, and cGAS-STING signaling might play an important role in the process.

## Supplementary Information

**Additional file 1: Figure S1.** The expression level and diagnostic value of AMICA1 in in LUAD and adjacent non-cancerous tissues. (A–C) The expression of AMICA1 in LUAD and adjacent non-cancerous tissues, including GSE32863 (T = 60, N = 60, P < 0.001), GSE75037 (T = 83, N = 83, P < 0.001) and TCGA (T = 49, N = 49, P < 0.001). (D–F) ROC curve of GSE32863 (ACU = 0.982, Sensitivity = 0.948, Specificity = 0.914), GSE75037 (AUC = 0.991, Sensitivity = 0.952, Specificity = 0.964) and TCGA database (AUC = 0.952, Sensitivity = 0.932, Specificity = 0.881).

**Additional file 2: Figure S2.** Univariate and multivariate Cox analysis of the expression of AMICA1 and LUAD clinical parameters. (A) Univariate Cox analysis. (B) Multivariate Cox analysis.

**Additional file 3: Figure S3.** Spearman’s correlation of AMICA1 with TILs, immunomodulators and chemokines (TISIDB). (A) Correlations between abundance of TILs and AMICA1 expression (plus the six TILs with the highest correlation). (B-D) Correlations between three kinds of immunomodulators and AMICA1 expression (plus the six immunomodulators with the highest correlation respectively). (E, F) Correlations between chemokines (or receptors) and AMICA1 expression (plus the six chemokines (or receptors) with the highest correlation respectively).

**Additional file 4: Figure S4.** The different expression of AMICA1 in infiltrating immune cells subtype of LUAD. (A, B) The expression of AMICA1 in early-stage LUAD tissue cells. (C, D) The expression of AMICA1 in infiltrating myeloid cells subtype of early-stage LUAD (LCs: Langerhans cells). (E, F) The expression of AMICA1 in advanced-stage LUAD tissue cells. (G, H) The expression of AMICA1 in infiltrating myeloid cells subtype of advanced-stage LUAD.

## Data Availability

The datasets used and/or analyzed during the current study are available from the corresponding author on reasonable request.

## Abbreviations

AMICA1: Adhesion molecule interacting with CXADR antigen 1
JAML: Junction Adhesion Molecule Like
TIICs: Tumor-infiltrating immune cells
OS: Overall survival rate
JAMs: Junctional adhesion molecules
CAR: Coxsackie–adenovirus receptor
HR: Hazard ratio
TILs: Tumor-infiltrating lymphocytes
cGAS: Cyclic GMP-AMP synthase
STING: Stimulator of interferon genes
MHC: Major histocompatibility complex
ECM: Extracellular matrix
