# Supplementary figures and images for: AMICA1 is a diagnostic and prognostic biomarker and induces immune cells infiltration by activating cGAS-STING signaling in lung adenocarcinoma
Source: Cancer Cell Int. 2022 Mar 5;22:111. doi: 10.1186/s12935-022-02517-x (PMC8897931; doi:10.1186/s12935-022-02517-x)

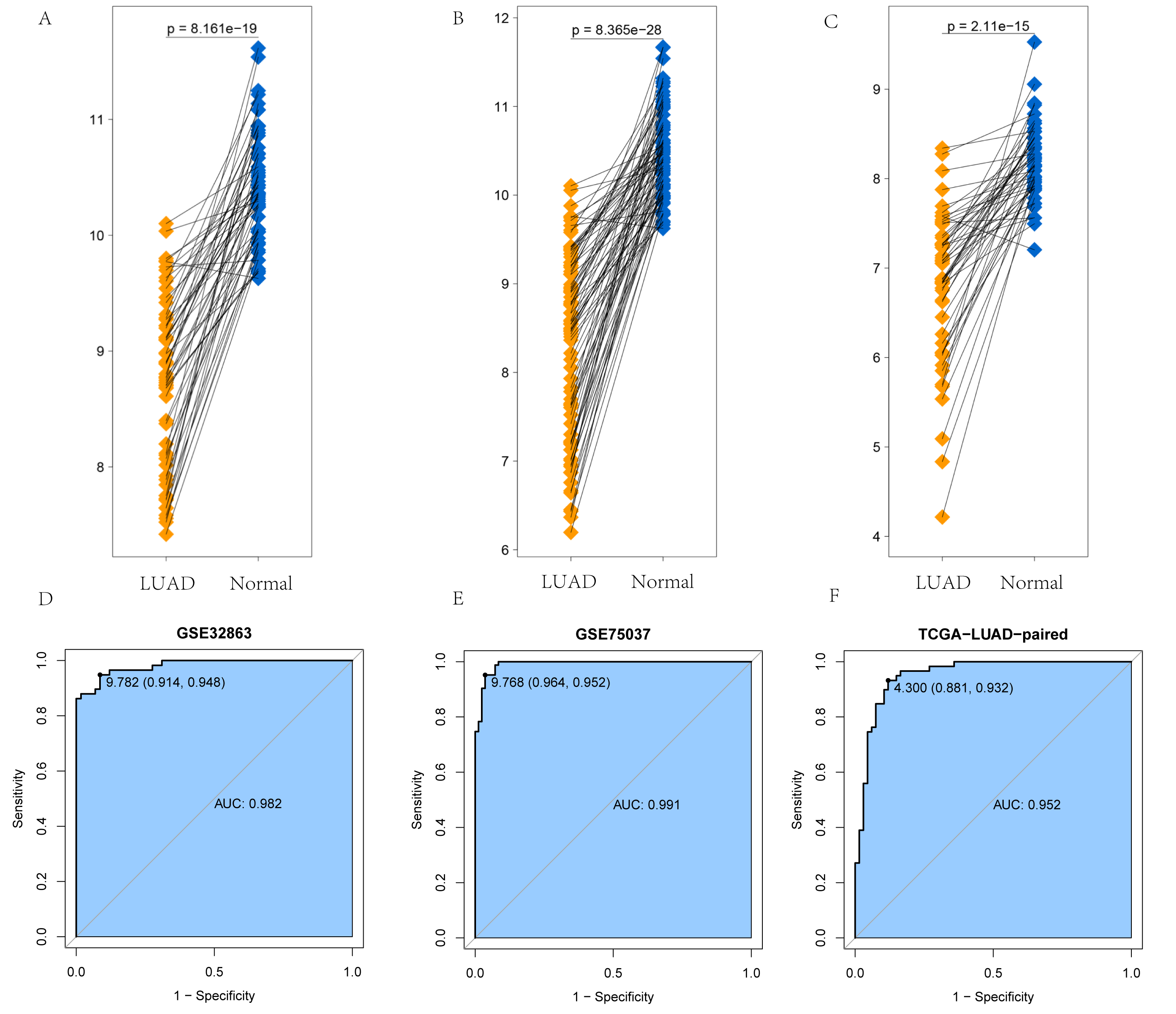

Supplement: Supplementary file 1 — Additional file 1: Figure S1. The expression level and diagnostic value of AMICA1 in in LUAD and adjacent non-cancerous tissues. (A–C) The expression of AMICA1 in LUAD and adjacent non-cancerous tissues, including GSE32863 (T = 60, N = 60, P < 0.001), GSE75037 (T = 83, N = 83, P < 0.001) and TCGA (T = 49, N = 49, P < 0.001). (D–F) ROC curve of GSE32863 (ACU = 0.982, Sensitivity = 0.948, Specificity = 0.914), GSE75037 (AUC = 0.991, Sensitivity = 0.952, Specificity = 0.964) and TCGA database (AUC = 0.952, Sensitivity = 0.932, Specificity = 0.881). [file 12935_2022_2517_MOESM1_ESM.tif]

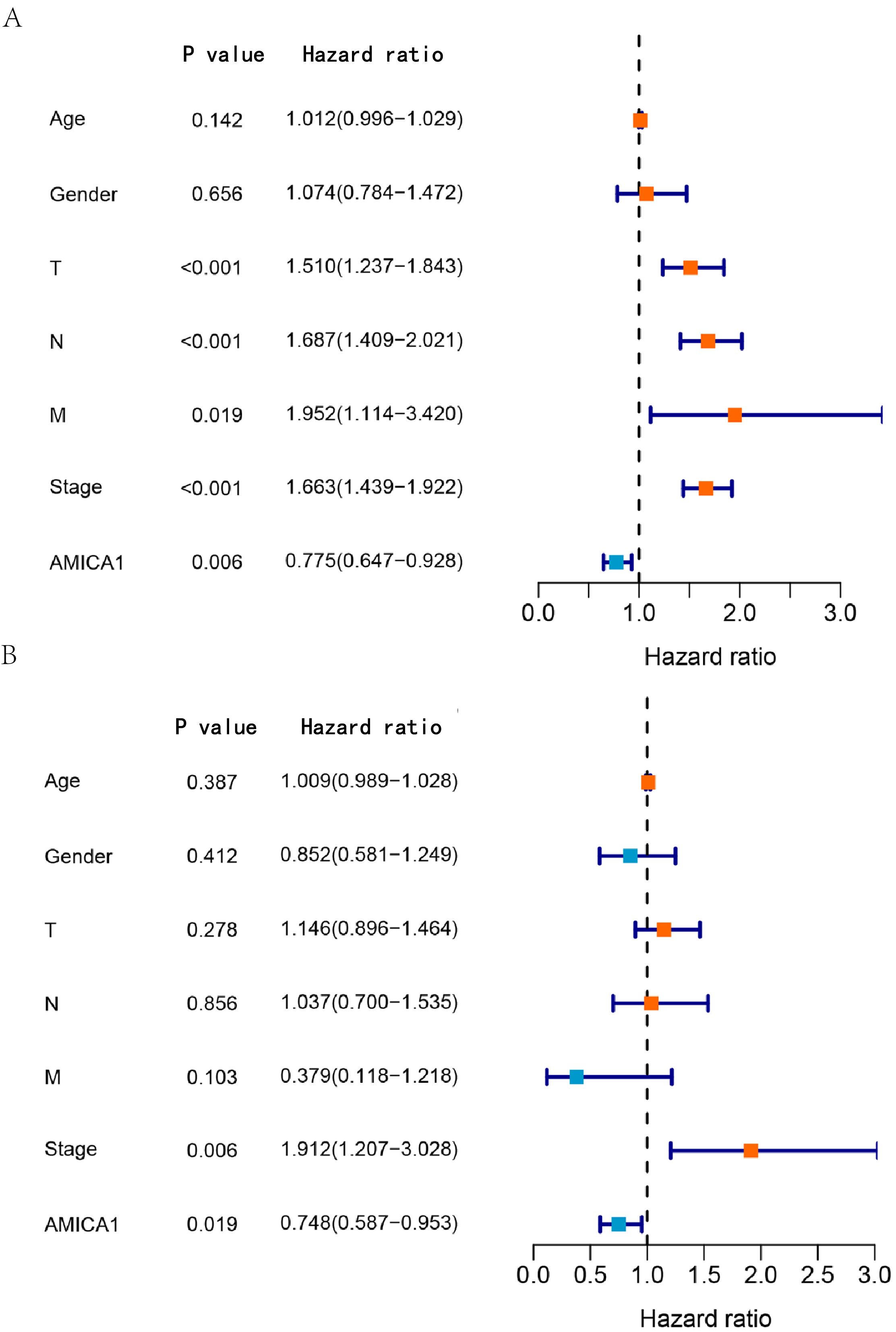

Supplement: Supplementary file 2 — Additional file 2: Figure S2. Univariate and multivariate Cox analysis of the expression of AMICA1 and LUAD clinical parameters. (A) Univariate Cox analysis. (B) Multivariate Cox analysis. [file 12935_2022_2517_MOESM2_ESM.tif]

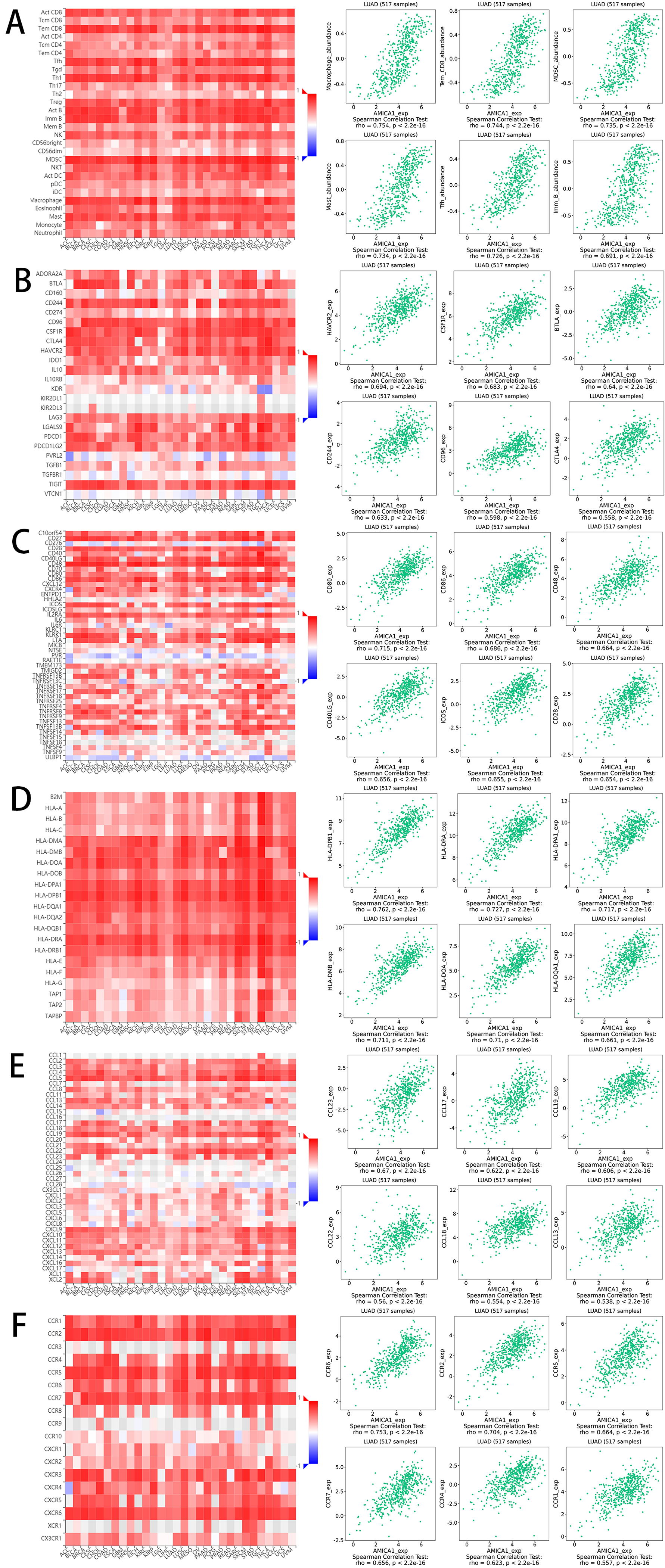

Supplement: Supplementary file 3 — Additional file 3: Figure S3. Spearman’s correlation of AMICA1 with TILs, immunomodulators and chemokines (TISIDB). (A) Correlations between abundance of TILs and AMICA1 expression (plus the six TILs with the highest correlation). (B-D) Correlations between three kinds of immunomodulators and AMICA1 expression (plus the six immunomodulators with the highest correlation respectively). (E, F) Correlations between chemokines (or receptors) and AMICA1 expression (plus the six chemokines (or receptors) with the highest correlation respectively). [file 12935_2022_2517_MOESM3_ESM.tiff]

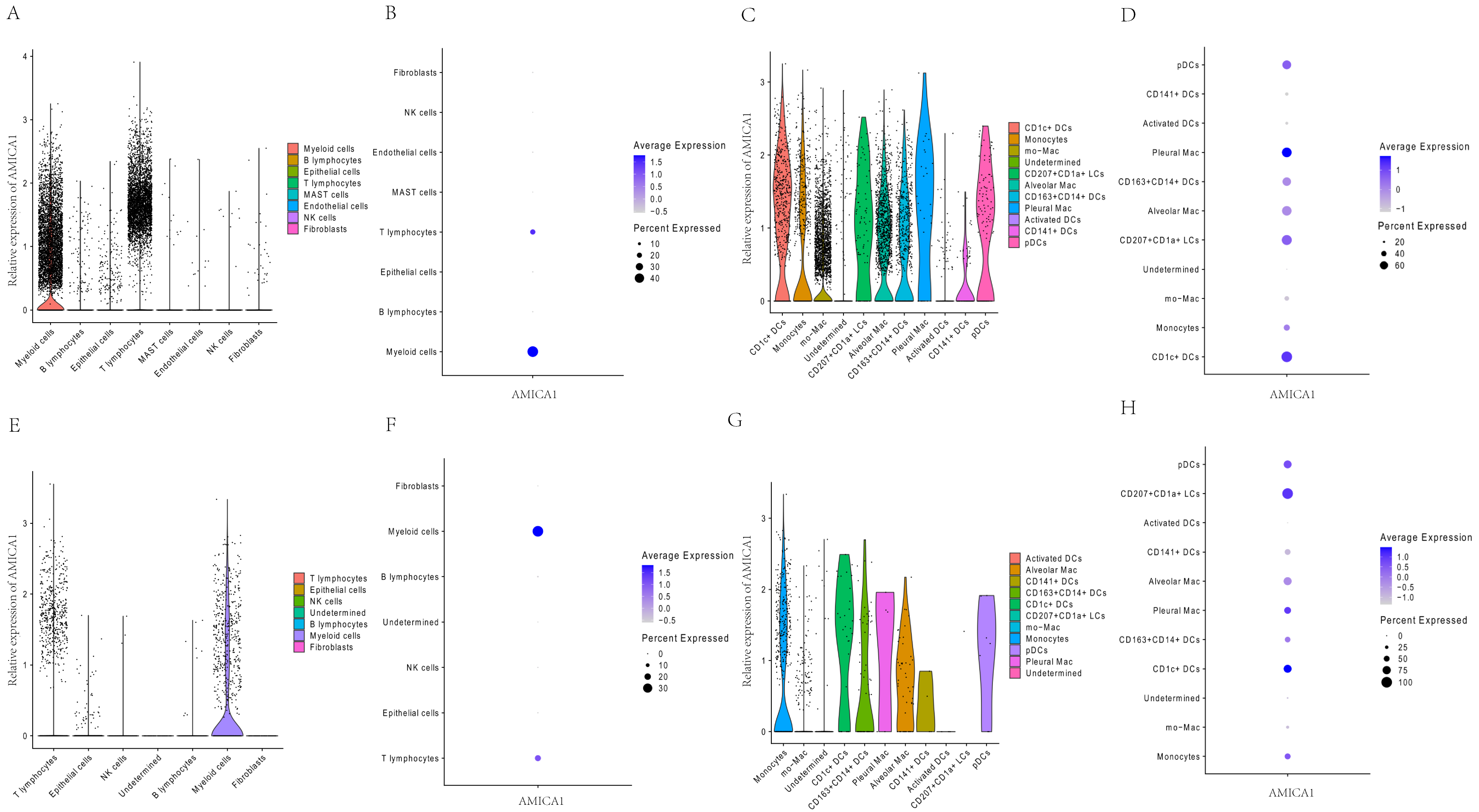

Supplement: Supplementary file 4 — Additional file 4: Figure S4. The different expression of AMICA1 in infiltrating immune cells subtype of LUAD. (A, B) The expression of AMICA1 in early-stage LUAD tissue cells. (C, D) The expression of AMICA1 in infiltrating myeloid cells subtype of early-stage LUAD (LCs: Langerhans cells). (E, F) The expression of AMICA1 in advanced-stage LUAD tissue cells. (G, H) The expression of AMICA1 in infiltrating myeloid cells subtype of advanced-stage LUAD. [file 12935_2022_2517_MOESM4_ESM.tif]
